# The Antimethanogenic Potentials of Plant Extracts: Their Yields and Phytochemical Compositions as Affected by Extractive Solvents

**DOI:** 10.3390/plants11233296

**Published:** 2022-11-29

**Authors:** Taofik Adam Ibrahim, Abubeker Hassen, Zeno Apostolides

**Affiliations:** 1 Department of Animal Sciences, University of Pretoria, Pretoria 0028, South Africa; 2 Department of Biochemistry, Genetics and Microbiology, University of Pretoria, Pretoria 0028, South Africa

**Keywords:** plant extracts, methanolic extractions, metabolomics, methane, in vitro

## Abstract

Plant phytochemicals are an important area of study in ruminant nutrition, primarily due to their antimethanogenic potentials. Plant extract yields, their bioactive compounds and antimethanogenic properties are largely dependent on the nature of the extractive solvents. This study evaluated the yields and phytochemical constituents of four plant extracts, as affected by the aqueous-methanolic (H_2_O-CH_3_OH) extraction and their antimethanogenic properties on the in vitro methane production. The plant extracts included *Aloe vera*, *Jatropha curcas*, *Moringa oleifera*, and *Piper betle* leaves with three levels of extractions (70, 85, and 100% CH_3_OH). The crude plant extract yields increased with the increasing amount of water. *M*. *oleifera* crude extracts yields (g/10 g) increased from 3.24 to 3.92, *A. vera*, (2.35 to 3.11) *J. curcas* (1.77 to 2.26), and *P. betle* (2.42 to 3.53). However, the identified and quantified metabolites showed differing degrees of solubility unique to their plant leaves in which they exist, while some of the metabolites were unaffected by the extraction solvents. The methane mitigating potentials of these extracts were evaluated as additives on *Eragrostis curvula* hay at a recommended rate of 50 mg kg^−1^ DM. The plant extracts exhibited antimethanogenic properties to various degrees, reducing (*p* < 0.05) in vitro methane production in the tested hay, *A. vera*, *J. curcas*, *M. oleifera* and *P. betle* reduced methane emission by 6.37–7.55%, 8.02–11.56%, 12.26–12.97, and 5.66–7.78 respectively compared to the control treatment. However, the antimethanogenic efficacy, gas production and organic matter digestibility of the plant extracts were unaffected by the extraction solvents. Metabolites, such as aloin A, aloin B and kaempferol (in *A. vera*), apigenin, catechin, epicatechin, kaempferol, tryptophan, procyanidins, vitexin-7-olate and isovitexin-7-olate (in *J. curcas*), alkaloid, kaempferol, quercetin, rutin and neochlorogenic acid (in *M. oleifera)* and apigenin-7,4′-diglucoside, *3-p*-coumaroylquinic acid, rutin, 2-methoxy-4-vinylphenol, dihydrocaffeic acid, and dihydrocoumaric acid (in *P. betle*) exhibited a methane reducing potential and hence, additional studies may be conducted to test the methane reducing properties of the individual metabolites as well as their combined forms. Plant extracts could be more promising, and hence, further study is necessary to explore other extraction methods, as well as the encapsulation of extracts for the improved delivery of core materials to the target sites and to enhance methane reducing properties. Furthermore, the use of 70% aqueous extraction on *M. oleifera* leaf is recommended for practical use due to the reduced cost of extractive solvents, the lower cost and availability of *Moringa* plants in South Africa, especially in Gauteng Province. Furthermore, 70% aqueous-methanolic extractions of *A. vera*, *J. curcas*, and *P. betle* are recommended for practical use in regions where they exist in abundance and are cost effective.

## 1. Introduction

One of the significant atmospheric methane contributors is ruminant animals. Methane emission contributes to the global warming effect and thus remains a topic of great interest, globally [1]. The contribution of enteric methane emission to total methane production from agricultural sources is quiet significant [2]. The need to find solutions to reduce the enteric methane emission without compromise to the performance and welfare of ruminants becomes inevitable. Many efforts, such as the supplementation of diets with concentrates [1] and lipids [3], use of probiotics and prebiotics [4], as well as the addition of medicinal plant extracts [5,6,7], have been used to mitigate enteric methane production. However, anti-methanogenic plant-based compounds, such as tannins, saponins, nitrates, and halogenated aliphatic hydrocarbons have been reported to limit some useful rumen microbial activities and reduce the animal performance, especially when added at higher doses, to achieve effective methane mitigation [7]. These adverse effects and toxicities may be averted through the use of multiple antimethanogenic compounds that may exert synergistic actions when used at appropriate doses, to inhibit the methanogenic archaea and other rumen protozoa that promote the methane production in an additive or complementary manner [8]. Some medicinal plants and their extracts, such as *Aloe vera*, *Jatropha curcas*, *Moringa oleifera*, and *Piper betle* have been reported to potentially modulate the rumen environment, reduce methane emissions and have antimicrobial activities [5,9]. This may be due to the combined activity of multiple bioactive compounds in the plant extracts. The abundance of medicinal plants/herbs in Africa has deepened the efforts of researchers to exploit plant-based compounds as potential natural alternatives to antibiotic growth promoters to enhance livestock productivity [10], including the decreased methane emission [5,11].

The characteristics, antioxidant, antimicrobial, and chemical constituents of *A. vera*, *J. curcas*, *M. oleifera*, and *P. betle* have been discussed in the previous study [5]. *A. vera* leaf is a rich source of anthraquinone, a phenolic compound that has stimulating effects on the bowels and antibiotic properties [12]. It contains saponins, which are soapy substances found in the gel and capable of cleansing. It exhibits antimicrobial activity against bacteria [12] and shows anti-inflammatory action, with a wide range of antimicrobial activity [13]. *J. curcas* is an ideal biodiesel crop in most arid areas of Asia, South America, and Africa, because of its high oil (43–61%) seed kernel [14]. Traditionally, *Jatropha* plants have been used to produce oil, soap, and medicinal compounds [14]. The various parts of the *M. oleifera* tree have been established as being good sources of glucosinolates, flavonoids, and phenolic acids [15,16]. Among the flavonoid compounds, flavonol glycosides of quercetin > kaempferol > isorhamnetin had been reported to be predominantly present in various parts of the tree, except in the roots and seeds [16]. *P. betle* is an evergreen perennial creeper [17]. Traditional healers used the betle leaf to treat halitosis, boils and abscesses, constipation, swelling of the gums, cuts, and injuries. In the South East Asia region, *P. betle* were among the plants that have been used to control caries and periodontal diseases [18] and to treat bad breath. Fathilah [18] reported that the crude aqueous extract of the *P. betle* leaves exhibits antibacterial activities towards *Streptococcus mitis*, *Streptococcus sanguis*, and *Actinomyces viscosus*, some of the early colonizers of dental plaque. Although the betle leaf has been tested in vitro for enteric methane emission in ruminants [5], the characteristics and properties of this plant make it a unique medicinal plant to further investigate.

The solvent extraction technique is significant in the determination of crude plant extract yield and the concentration of bioactive compounds in plant extracts. Increasing the efficiency of solvents in the extraction of plant-based compounds in medicinal plants, requires the choice of the right combination of extraction medium [19,20,21]. This may be because some bioactive compounds in plant materials are relatively hydrophobic and others hydrophilic. It has also been stated that no universal extraction method is ideal and each extraction procedure is unique to the targeted plant compounds [21]. Water is a universal solvent, used to extract plant products with antimicrobial activity. Though plant extracts from organic solvents give more consistent antimicrobial activity, compared to water extract, water-soluble phenolics are mostly important as antioxidant compounds [22]. In order to extract the different phenolic compounds from plants with a high degree of accuracy, various solvents of different polarities have to be used [23]. Studies had shown that highly polar solvents, such as methanol, have a high effectiveness as antioxidants [24] and antimicrobial resistance [5]. However, the yields/concentrations of biologically active compounds in plant extracts could be increased further by the addition of a more polar solvent, such as water. The higher concentrations of bioactive flavonoid compounds were recorded with aqueous-alcohol (30:70), due to its higher polarity than pure alcohol [25].

Solvents with different polarities have been used to obtain extracts in plant-based materials and different results concerning the yields and antimicrobial efficacy were reported. Hence, this study investigated the effect of an extraction efficiency of various aqueous-methanol concentrations on yields, phytochemical constituents, and the antimethanogenic potential of *A. vera*, *J. curcas*, *M. oleifera* and *P. betle* leaf extracts. It is hypothesized that the use of different proportions (70%, 85, and 100% methanol) of aqueous-methanol solvent extractions may have a useful effect in improving the crude plant yields, phytochemical concentrations, and subsequently the methane mitigation potential of *A. vera*, *J. carcas*, *M. oleifera*, and *P. betle* leaf extracts. 

## 2. Results and Discussion

### 2.1. The Yield of Plant Crude Extracts

The crude extract yields (Table 1) from the medicinal plants were influenced by the treatment solvents with consistent trends and increased (*p* < 0.05) with a decrease in the amount of methanol (CH_3_OH) solvents, replaced by an equivalent amount of distilled water (H_2_O) in the mixture for all of the plant samples. The yields of the *M*. *oleifera* crude extracts increased from 3.24 to 3.92 g/10 g, *A. vera* (2.35 to 3.11 g/10 g), *J. curcas* (1.77 to 2.26 g/10 g), and *P. betle* (2.42 to 3.53 g/10 g). These results are within the average value of the 30% extract yields of green tea earlier reported [26]. The extraction procedures and solvent type play a critical role in the determination of crude extract yields and the concentration of bioactive compounds in medicinal plants [21]. The results of this study show that the four plants used in the study are probably water-soluble, with *M. oleifera* having more relatively hydrophilic phytochemicals than *A. vera*, *J. curcas*, and *P. betle*. The increase in yield of plant crude extracts with increased distilled water in the solvent mixtures could mean that most bioactive compounds in the plant leaves are hydrophilic and could easily leach out, preferentially, in the presence of polar solvents. To increase the efficiency of the extraction in medicinal plants, the use of solvents with different polarities is critical [27]. The combination of CH_3_OH and H_2_O as extraction solvents in this study could have softened the plant samples in the mixture better than pure CH_3_OH and promoted the rapid physiological absorption of the extracts due to the higher polarity of H_2_O [21,28,29].

### 2.2. Phytochemical Identification and the Concentration of Plant Metabolites

#### 2.2.1. *Aloe vera*

The study reveals the presence of bioactive compounds in the aqueous-methanolic extraction of *A. vera* with the detailed characterization of the identified compounds (Table 2). The chromatogram representations of the analysed phytochemicals are contained in Figures SA1–SA3. The concentrations (mg L^−1^) of aloesin, nataloin A, nataloin B, 10-hydroxyaloin A, 10-hydroxyaloin B, and caffeoyl ester of aloesin were higher at 100% methanol extraction and lower at 70% methanol extraction, indicating they were relatively less water-soluble. While kaempferol-7-*O*-glucoside and 3-*p*-coumaroylquinic acid were relatively hydrophilic, the extraction solvents had no clear effect on the abundance of aloin A, aloin B, and aloe emodins as the concentrations of these metabolites were likely affected by the relative solubility behaviour of other metabolites in the crude plant extracts. Aloin A, aloin B, and aloe emodin have been reported in a few aloe species crude extracts [30,31,32,33]. Kaempferol, 10-hydroxyaloin A and B, and caffeoyl ester of aloesin and aloesin have also been identified in the solvent extracts of the aloe species [30,31]. Quispe et al. [28] reported the presence of aloin A, aloin B, aloe emodin diglucoside, 10-hydroxyaloin A, 6-malonylnataloin A (nataloin A), and caffeoyl ester of aloesin in *A. vera* aqueous extracts. Additionally, 6-malonylnataloin A and B, aloinoside A/B, and aloeresin have been identified in three to six species of aloe [30]. From the few identified metabolites in *A. vera*, kaempferol-7-*O*-glucoside and 3-*p*-coumaroylquinic acid were relatively water-soluble and had a direct relationship with the crude plant extracts (in Table 1) while other metabolites were either hydrophobic or unclearly affected by the extraction solvents. The possible reason is that there are other unidentified metabolites for which a relative solubility is yet to be understood. However, this study focused more on the metabolites in crude plant extracts capable of reducing the enteric methane production and how they are influenced by the extraction of solvents. The principal constituent of the compounds in *Aloe vera* is anthraquinone/anthrone with a broad spectrum of biological activities, such as anticancer, anti-inflammatory, antimicrobial, diuretic, vasorelaxing, and phytoestrogen indicating their possible clinical use in several diseases [34].

#### 2.2.2. *Jatropha curcas*

The principal component of the phenolic compounds identified in the *J. curcas* leaf extract (Table 3) in this study are flavonoids with a few procyanidin compounds. The chromatograms showing the various peaks of the identified phytochemicals in *J. curcas* are contained in the Figures SJ1–SJ3. The concentration (mg L^−1^) of vitexin-7-olate, isovitexin-7-olate, kaempferol-7-*O*-glucoside, apigenin-7-*O*-rutinoside, and apigenin-6-*C*-arabinosyl-8-*C*-arabinoside were not affected by the extraction solvents. The concentration of catechin, epicatechin, and procyanidins are affected by the extraction solvents, with the greater concentration at 100% methanol extraction, and the lowest at 70% methanol extraction. However, the difference in the concentration of catechin, tryptophan, and procyanidin dimer B1 is relatively small. Catechin and epicatechin are phytochemicals mostly reported in *Jatropha,* in recent times; they were identified in methanolic extracts of *J. curcas* and *J. cinerea* kernel meal [29], catechin in the methanol extracts of the *J. curcas* leaf [35], and catechin and its derivatives were reported in the *J. macrantha* stems [36]. Catechin and epicatechin have been reported to exhibit methane reducing properties [29]. Vitexin-7-olate and isovitexin-7-olate have been detected in the ethanolic extracts of the *J. curcas* leaves [14] and the crude leaf extracts of *J. gossipifolia* [37]. Other similar compounds that have been reported in *Jatropha,* include apigenin and its glycosides in *J. platyphylla* [38] and kaempferol in *J. curcas* [39], however, procyanidin dimers B1, B2 and procyanidin trimer C1 and C2 have not been reported in any species of the *Jatropha* plants. Most of the plant compounds identified in *J. curcus* have therapeutic activities. For example, apigenin has anticancer, anti-depressing, antidiabetic, and health promoting properties with learning and memory enhancing activities [40], while procyanidins reportedly exhibited antioxidant, anticancer, anti-inflammatory, immunosuppressive, and antiallergy activities with protection against chronic diseases and metabolic disorders [41,42]. Vitexin and isovitexin both possess antioxidant, anti-inflammatory, antidiabetic, anticancer, and neuroprotective properties [43].

#### 2.2.3. *Moringa oleifera*

The phenolic profile of the *M. oleifera* leaf extract shown in Table 4 consists of phenolic, flavonoid, and polyamine alkaloid compounds. The chromatogram illustrations of the quantified phytochemicals in this study are contained in Figures SM1–SM3. These include chlorogenic acid, neochlorogenic acid, 3-*p*-coumaroylquinic acid, feruloylquinic acid, rutin, quercetins, kaempferols, and alkaloids. The concentrations of these metabolites in moringa leaf extracts were not clearly affected by the extraction solvents, except the alkaloids. The principal compound present in the *M. oleifera* leaf extract observed in this study was the flavonoids, which validated a previous report [15]. A number of similar quercetin and kaempferol derivatives and isomers, such as kaempferol-3-*O*-glucoside, kaempferol-3-*O*-rutinoside, kaempferol-3-*O*-acetyl-glucoside, and rutin, have been previously reported in *M. oleifera* leaves [15,44], while some flavonoids, such as kaempferol and its derivatives [45], have been detected. Chlorogenic acid (5-caffeoylquinic acid) and neochlorogenic acid (3-caffeoylquinic acid) have been reported to be present in *M. oleifera* leaf extracts [15,46]. Feruloylquinic acid, quercetin-3-*O*-acetyl-glucoside (quercetin-3-acetyl-glucoside), cinnamoylquinic acid (3-*p*-coumaroylquinic acid), kaempferol-3-*O*-glucoside, and quercetin-3-*O*-hexoside and their isomers have also been reported [46]. A phytochemical screening study by Onyekaba et al. [47] revealed that flavonoids, terpenoids, phenolics, and alkaloids characterizing the *M. oleifera* leaf extracts possessed a marked antibacterial potential against *E. coli* and *Pseudomonas aeruginosa*, and this strong antibacterial activity probably needs to be explored in the methane studies. This study also confirms the presence of polyamine alkaloids in *M. oleifera*, which is also in tandem with the earlier findings of Leone et al. [48]. Alkaloids have muscle relaxant, antioxidant, anticancer, antimicrobial and amoebicidal properties [49]. Kaempferol has been widely used in treating cancer, cardiovascular diseases, metabolic complications, and neurological disorders [50]. Quercetin is another flavonoid with a wide variety of biological activities, such as antioxidant, broad-spectrum antibacterial and antiparasitic properties, cardiovascular protection, anti-immunosuppression treatment, and reduce the toxicity of mycotoxins [51], while rutin has been reported as a strong antioxidant with cancer preventive properties [52]. Chlorogenic acid has liver and kidney protective properties, antioxidant, anti-tumor, antibacterial, anti-inflammatory, as well as regulation of glucose and the lipid metabolism [53].

#### 2.2.4. *Piper betle*

The phenolic profile of the *P. betle* leaf extract is presented in Table 5 while the chromatograms illustrating the analysed metabolites in *P. betle* are contained in Figures SP1–SP3. In contrast to *Jatropha curcas*, the principal components of the phenolic compounds in the *P. betle* leaf extract identified in this study are phenolic acids with a few flavonoids. Phenolic acids consist of coumaric acid and its derivatives and compounds of caffeic acid, while the flavonoids are rutin and apigenin-7,4′-diglucoside. The relative metabolite concentrations (mg L^−1^) of 100% methanol extraction were low for coumaric acid, 3-*p*-coumaroylquinic acid, dihydrocaffeic acid, and dihydrocoumaric acid while 70% and 85% methanol extractions recorded higher concentrations, indicating that these metabolites were relatively more water-soluble. On a contrary, rutin, apigenin, and methoxy-4-vinylphenol are relatively less water-soluble and had a higher concentration at 100% methanol extraction. Similar compounds were noted by Lee et al. [54], who investigated the antimicrobial, antifungal, and antioxidant activities of the *P. betle* leaf. Rutin and coumaric acid (chavibetol) have been reported to be present in chloroform extracts of the *P. betle* leaf [55], while Purba and Paengkoum [56] identified compounds, such as rutin, coumaric acid, caffeic acid, and apigenin in different solvent extracts of *P. betle*. Caffeic acid is abundant in coffee and tea with good antioxidant, anti-inflammatory, anticancer, and neuroprotective properties [57], while coumaric acid possesses bioactivities, such as antioxidant, anti-inflammatory, analgesic, and antimicrobial, prevent liver damage and exhibit an amoebostatic activity against *Entamoeba histolytica* [58].

### 2.3. In Vitro Organic Matter Fermentation

**Figure 1:**
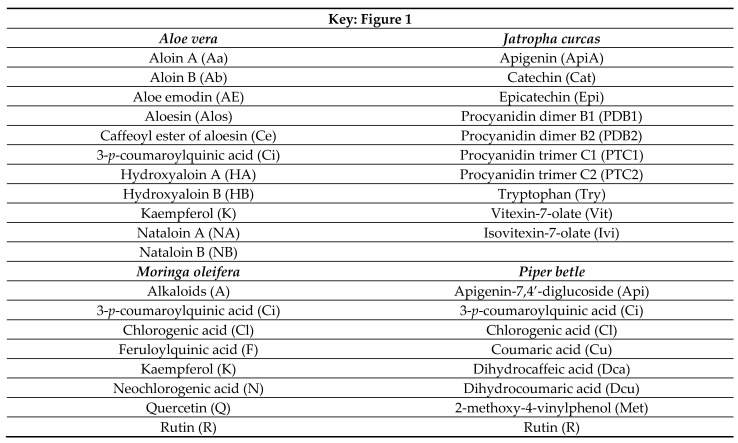

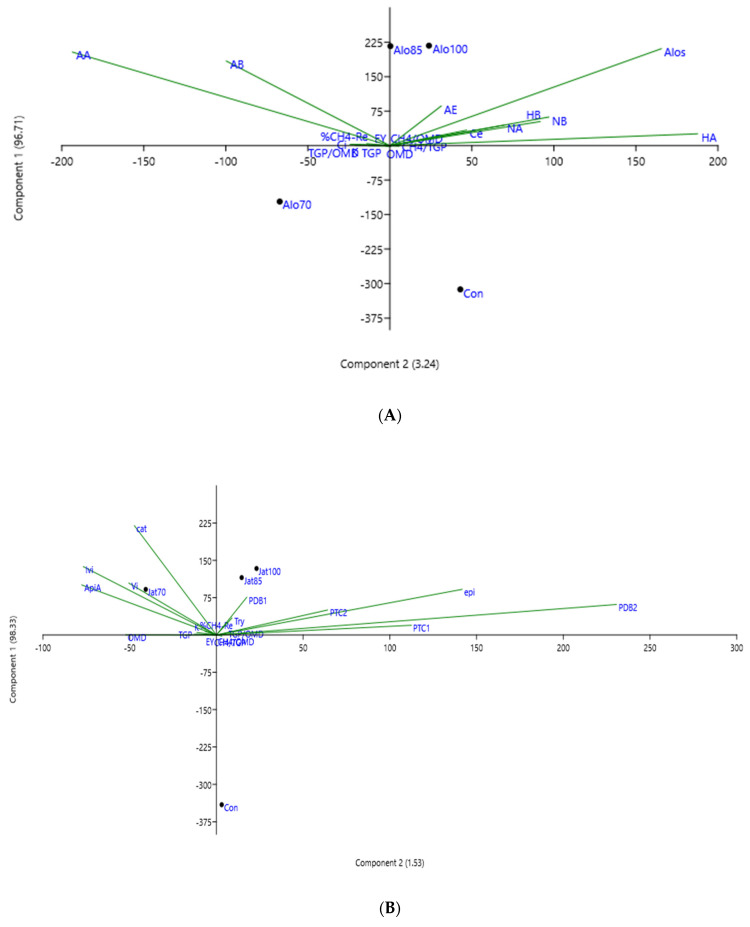

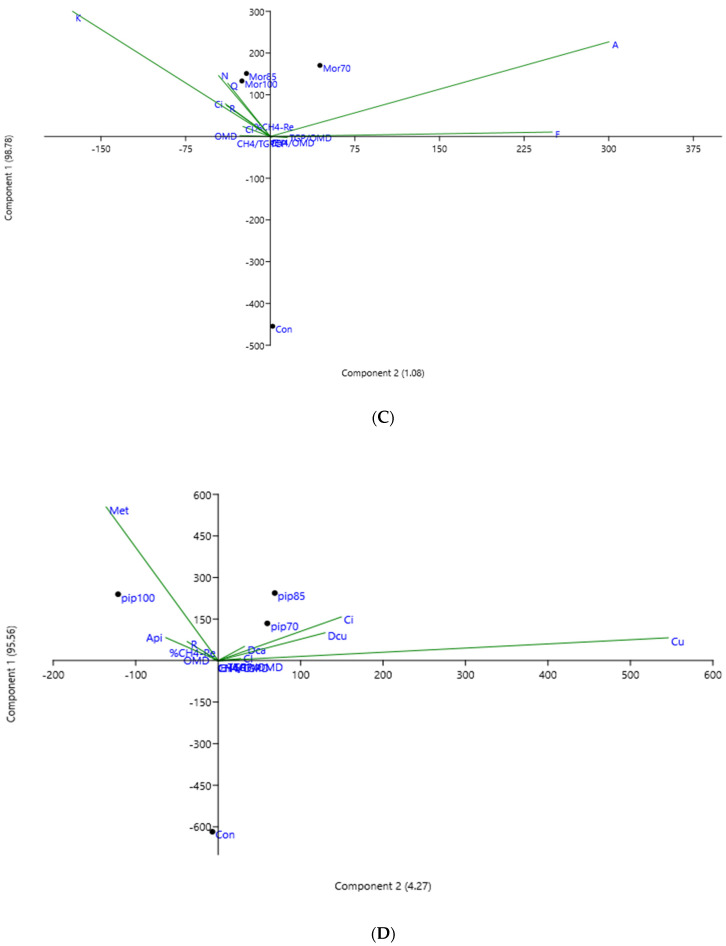
Principal component analysis plot 1 vs. plot 2 of all fermentation parameters of *Eragrostis curvula* hay fermented with three different aqueous-methanol (70, 85, and 100%) extractions of *Aloe vera* (**A**), *Jatropha curcas* (**B**), *Moringa oleifera* (**C**), and *Piper betle* (**D**) leaf extracts.

This section evaluated the antimethanogenic potentials of three different combinations (70, 85, and 100%) of two extractive solvents (CH_3_OH and H_2_O) of *A. vera*, *J. curcas*, *M. oleifera*, and *P. betle* on *Eragrostis curvula* hay. The substrate, *E. curvula* hay, used in this study had 92% DM while crude protein, ash, and ether extract, respectively, contained 5.12, 9.1, and 1.3% of DM. The NDF, ADF, and ADL contents were 75.5, 44.5, and 8.1% of DM, respectively. The high content of NDF, ADF, and ADL of the feed causes an increase in the amount of methane (CH_4_) formed in the rumen fermentation. The CH_4_ emission, total gas production and organic matter digestibility of *E. curvula* hay are presented in Table 6, while the principal component analysis (PCA) of the CH_4_ emission, total gas production, and organic matter digestibility of *E. curvula* hay fermented with crude plant extracts of *A. vera*, *J. curcas*, *M. oleifera*, and *P. betle* are presented in Figure 1 while the principal component loadings and correlation results are illustrated in Tables S1 and S2 respectively. The plant extracts reduced (*p* < 0.05) in vitro the methane production in the tested hay. *Aloe vera*, *Jatropha curcas*, *Moringa oleifera*, and *Piper betle* reduced the methane emission by 6.37–7.55%, 8.02–11.56%, 12.26–12.97, and 5.66–7.78, respectively, compared to the control treatment. However, the extraction solvents did not affect the antimethanogenic efficacy, gas production, and organic matter digestibility of the crude plant extracts. The aqueous-methanolic extractive solvents were observed to increase (*p* < 0.05) the crude extract yields of the test leaves, compared to the pure methanolic extraction (Table 1); however, these yields did not affect the methane reducing potentials of the leaf extracts. This may be due to the little variation in the physical and chemical activities of the two combined solvents (CH_3_OH and H_2_O). The use of different solvents to obtain extracts in plants increased the variation in bioactive compounds in the crude extracts, compared to the proportionate mixing of the two solvents [59]. According to Sabandar et al. [60], the variation in the antimicrobial activity of the *J. unicostata* extract was higher between the solvents chloroform and methanol, compared to their proportionate combinations. The little intra-variation in the solvent properties, the little/no difference in the concentration of the methane reducing metabolites, and the patterns in which the metabolites in the crude plant extracts affect fermentation could probably be responsible for the non-significant variation in the methane production between extraction solvents. For example, in the PCAs of *A. vera*, aloin A, aloin B, and kaempferol were metabolites associated with the CH_4_ reduction. The 70% methanol extraction of *Aloe vera* had a higher concentration of kaempferol but a lower concentration of aloin A and aloin B, while the 85% and 100% methanol extractions had a higher concentration of aloin A and aloin B, but a lower kaempferol (Table 2). In *J. curcas* (Figure 1B), catechin, apigenin, kaempferol, vitexin-7-olate, and isovitexin-7-olate were more associated with methane reduction in the 70% methanol extraction, while epicatechin, tryptophan, procyanidin dimer B1, and procyanidin trimer C2 were more associated with methane reduction in 85% and 100% methanol extraction. Furthermore, the differences in the concentrations (Table 3) of catechin, vitexin-7-olate, isovitexin-7olate, apigenin, kaempferol, and procyanidin dimer B1 in the crude plant extracts of *J. curcas,* were very small and not affected by the extraction solvents. In *M. oleifera* (Figure 1C), kaempferol, quercetin, rutin, neochlorogenic acid, and 3-*p*-coumaroylquinic acid had more associative effects for the CH_4_ reduction in the 85% and 100% methanol extractions while alkaloid was more associated with the CH_4_ reduction in the 70% methanol extraction. In addition, the percentage difference in the concentrations of these antimethagenic metabolites (Table 4) in the crude plant extracts was little and not influenced by the extraction solvents. In *P. betle* (Figure 1D), apigenin, rutin and 2-metoxy-4-vinylphenol were more associated with the CH_4_ reduction in 100% methanol extraction while *3-p*-coumaroylquinic acid, dihydroxycaffeic acid, and dihydroxycoumaric acid were more associated with CH_4_ mitigation in the 70% and 85% methanol extractions. While the concentrations of rutin, apigenin-7,4′-diglucoside, and 2-metoxy-4-vinylphenol were low in the crude plant extract with the 70% methanol extraction, this could probably be balanced with other methane reducing metabolites which were relatively higher in concentration. The primary objective of identifying the metabolites in the crude plants’ extracts is to establish or validate their antimethanogenic potentials. Some of the metabolites of *A. vera*, *J. curcas*, *M. oleifera*, and *P. betle* extracts identified in this study have been reported to possess antimicrobial activities with good antimethanogenic properties. The presence of alkaloids, flavonoids, and phenols in plant extracts had been attributable to reducing the enteric methane in ruminants [5,61]. Furthermore, alkaloids, due to their bitter tastes [62,63] in moringa, could create an undesirable condition for some ruminal microbes. Flavonoids have been evaluated for rumen methanogenesis [64]. Oskoueian et al. [65] reported that the inclusion of flavone, myricetin, naringin, rutin, quercetin, or kaempferol decreased the in-vitro methane emission by 5 to 9 mL g^−1^ DM while catechin reduced methane production both in vitro [66] and in vivo [67]. All the flavonoids, when fed at 0.2 g kg^−1^ DM, noticeably decreased the relative abundances of the hydrogenotrophic methanogens. Flavonoids have been reported to directly suppress methanogens [65,68] and also likely act as H_2_ sinks via the cleavage of the ring structures (e.g., catechin) and the reductive dihydroxylation [66]. The anthraquinones and flavonoids [12] in *A. vera* had been reported to exhibit strong antimicrobial activities against bacteria and fungi, while an early study revealed that acetone and methanolic extraction of aloe [69] and the pure methanolic extraction of aloe [5], reduced in vitro the methane production. The aqueous extraction of *P. betle* showed the presence of alkaloids, phenolic compounds, and alcoholic compounds with a good antimicrobial function [70] and effective inhibitory action against microorganisms [71]. According to Santra et al. [72], the ethanolic extracts of *J. gossipifolia* reduced in vitro the methane production by 31% and also inhibited the growth of the rumen protozoal population, due to the presence of phenolic compounds in the extracts. The antimethanogenic property of 3-*p*-coumaroylquinic acid, dihydroxycaffeic acid, and dihydroxycoumaric acid had been previously reported in compounds with similar chemical activities, such as cinnamic acid, caffeic acid, and coumaric acid, respectively [73]. The use of 2 mM of cinnamic acid, caffeic acid, and coumaric acid decreased the in vitro methane production without reducing the organic matter digestion [73], while the use of caffeic acid reduced the in vitro methane production [74]. The reduction in enteric methane (*p* < 0.05) observed in this study for all of the treatment plant extracts is consistent with previous findings [5], except for *P. betle,* which increased methane in a previous study [5] against the reduction (*p* < 0.05), observed in this study. Plant extracts have a complex blend of bioactive components with many variations in their composition, due to biological factors, production techniques, and storage conditions [63], while parameters that affect the efficacy of the plant extracts are genetic variations of the plant, the age of the plant, dosage, extraction technique, harvest time, and compatibility with other ingredients [62]. All these conditions will probably impose variations on the parameters of interest.

## 3. Materials and Methods

### 3.1. Study Area and Collection of the Plant Materials

**Figure 2:**
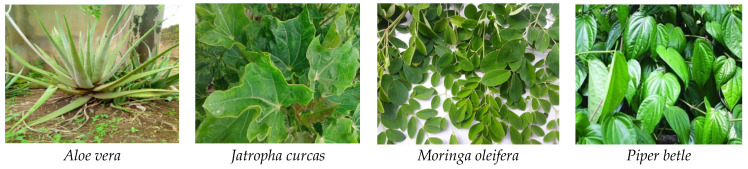
Plant leaves from which the crude plant extracts collected.

The study was conducted at the departmental laboratory of the Department of Animal and Wildlife Sciences, University of Pretoria, South Africa, following its ethical approval (NAS336/2019). The *Moringa oleifera* leaves (A11NA) used in this study were harvested fresh from growing and blooming trees at Lefakong farm in Pretoria North at 399 Thaba ya Batho Boplaas, South Africa, while *Piper betle* leaves (cultivar Marakodi) were collected from a farm in Durban at Tongaat Kwazulunata, South Africa. *Jatropha curcas* (IARJAT-S1) and *Aloe vera* (Taxon A) leaves were imported from Nigeria and harvested at full maturity in Kaduna state, a northwestern region in Nigeria, with the permission of the Department of Agriculture (P0095290), South Africa. Samples were collected from multiple representative plants of the same species (Figure 2).

### 3.2. Methanolic Extraction

*Moringa oleifera*, *Jatropha curcas*, *Aloe vera*, and *Piper betle* aqueous-methanolic extracts were prepared using methanol (CH_3_OH) at 70, 85, and 100% concentrations as a modification to the previous method [5]. The plant materials were first freeze-dried for 96 h and stored in plastic bags pending further use. Dried samples of each plant leaf were milled through a 1 mm sieve and extracted by dissolving 10 g of milled dried leaf material into a 300 mL extraction bottle containing 200 mL (1:20 *w*/*v*) aqueous methanol [70% (3 mL H_2_O: 7 mL CH_3_OH), 85% (1.5 mL H_2_O: 8.5 mL CH_3_OH), and 100% (CH_3_OH only)]. The extraction bottles were arranged into an Incoshake incubator and agitated at 130 rpm and 20 °C for 96 h. Extracts from each bottle were filtered by squeezing through a sieve with a 150 μm aperture. The filtrate was placed in a fume cubicle for 24 h until partially dried. The semi-dried extracts were freeze-dried for 72 h. All freeze-dried extracts were stored in plastic bottles at 4 °C until further use.

### 3.3. Ultra-Performance Liquid Chromatography—Mass Spectrometry (UPLC–MS) Analysis of the Bioactive Molecules in Plant Extracts

The phytochemical identification of the crude extracts was carried out using ultra-performance liquid chromatography–mass spectrometry (UPLC–MS). A Waters Synapt G2 Quadrupole time-of-flight (QTOF) mass spectrometer (MS) connected to a Waters Acquity ultra-performance liquid chromatograph (UPLC) (Waters, Milford, MA, USA) was used for the high-resolution UPLC–MS analysis. Electrospray ionization was used in negative mode with a cone voltage of 15 V, a desolvation temperature of 275 °C, a desolvation gas at 650 L/h, and the rest of the MS settings optimized for the best resolution and sensitivity. Data were acquired by scanning from m/z 150 to 1500 m/z in resolution mode, as well as in the MSE mode. In the MSE mode, two channels of MS data were acquired, one at a low collision energy (4 V) and the second using a collision energy ramp (40−100 V), to obtain the fragmentation data. Leucine enkaphalin was used as the lock mass (reference mass) for the accurate mass determination, and the instrument was calibrated with sodium formate. The separation was achieved on a Waters HSS T3, 2.1 × 100 mm, 1.7 μm column. An injection volume of 2 μL was used, and the mobile phase consisted of 0.1% formic acid (solvent A) and acetonitrile containing 0.1% formic acid as solvent B. The gradient started at 100% solvent A for 1 min and changed to 28% B over 22 min in a linear way. It then went to 40% B over 50 s and a wash step of 1.5 min at 100% B, followed by re-equilibration to the initial conditions for 4 min. The flow rate was 0.3 mL/min, and the column temperature was maintained at 55 °C. The identification was performed, based on its measured mass, compared to the theoretical mass (<5 ppm), the molecular formula and the characteristic fragments for each compound, finding the differences and similarities between the samples analysed. The library database was used to identify compounds present in the crude extracts. Data was reprocessed using MSDIAL and MSFINDER (RIKEN Center for Sustainable Resource Science: Metabolome Informatics Research Team, Kanagawa, Japan) in order to utilise the fragmentation data contained in the Waters MSe acquisition [75]. The peak height data from MSDial was used to determine the mean abundance of the identified metabolites in [76].

### 3.4. Buffer Mineral Solution, Collection of the Rumen Fluid from Donor Steer and the In Vitro Gas Production

The in vitro gas production studies were carried out following the procedure of Menke et al. [77], with the modifications detailed in Adejoro and Hassen [78]. The prepared buffer solution was preserved in a water bath at 39 °C and constantly purged with CO_2_ until the solution turned colourless. The rumen fluid was collected from three rumen-cannulated Holstein breed of cattle fed Lucerne hay *(Medicago sativa) ad libitum*. A 40 mL prepared solution of rumen fluid was used to incubate 400 mg of the substrate in a 120 mL in vitro bottle in triplicates and four successful runs known as biological replicates were conducted. Gas pressure was taken at 2, 4, 8, 12, 24, and 48 h after the commencement of the incubation, while gas samples were taken inside Hamilton syringes for the analysis of methane concentration. Three blanks were included to correct the methane produced from the inoculum in each run. Methane concentration was analysed with gas chromatography (8610C BTU Gas Analyser GC System; SRI Instruments GmbH, Bad Honnef, Germany). The GC was pre-equipped with a solenoid column, packed with silica gel and a flame ionization detector (FID). Methane concentration values were related to the total gas production, in order to estimate its concentration. Methane concentration was subsequently converted to mass values [78]. Eragrostis hay of known chemical composition was used as a substrate and incubated with the crude extracts of four medicinal plants prepared using different percentages of methanol/aqueous solvent extraction (70, 85, and 100%). Plant extracts were reconstituted in distilled water and added to the test the feed at 50 mg kg^−1^ DM. For the control treatment, there was an equal amount of distilled water without plant extracts.

### 3.5. Determination of the In Vitro Organic Matter Digestibility (IVOMD)

Extracts of *Aloe vera*, *Jatropha curcas*, *Moringa oleifera*, and *Piper betle* were evaluated as additives to 1 mm particle size Eragrostis hay substrate using the IVOMD procedure [5]. Briefly, the first stage involved a 48 h rumen degradation phase, followed immediately by another 48 h acid-pepsin digestion phase. During the first phase, 200 mg of the feed samples were incubated in triplicate under anaerobic conditions with 20 mL of rumen liquor for 48 h at 39 °C with the inclusion of blanks and standards in every batch of incubation. This was followed by a 48 h acid-pepsin digestion phase at 39 °C under anaerobic conditions. Following 96 h of incubation, the residual plant materials were collected and oven-dried at 105 °C for 18 h. The ash contents were determined by combustion in a muffle furnace at 250 °C for 2 h and later at 600 °C for 4 h, and the in vitro organic matter digestion was estimated.

### 3.6. Chemical Analyses

The feed sample, *Eragrostis curvula* hay, was analysed for the dry matter (DM) and total ash [79]. The ether extract was determined using the ether extraction in the Tecator Soxtec (HT6) system [80]. Neutral detergent fibre (NDF), acid detergent fibre (ADF) and acid detergent lignin (ADL) contents were determined using an ANKOM200/220 fibre analyser (ANKOM Technology, Fairport, NY, USA), as described [81], nitrogen [82] (FP2000 Nitrogen/Protein Analyser, Leco Instrumente GmbH, Kirchheim, Germany), and the crude protein was obtained by multiplying nitrogen by 6.25.

### 3.7. Statistical Analysis

The crude extract yield of the plant samples was evaluated in triplicate, and the data collected were analysed using the GLM procedure of SPSS (version 20) with the model Yij = µ + Tj + eij. where Yij is the mean of the individual observations (crude extract yield), μ is the overall mean, Tj is the treatment effect (extraction solvent), and eij is the residual error. The means were separated using the Tukey test, and the significance was declared at *p* < 0.05. For the in vitro gas production study, individual bottles within each run served as analytical replicates, while each run represented a statistical replicate. Data were analysed using the GLM procedure in SPSS software (version 20) with the model Yij = µ+ Bi + Tj + eij. where Yij = mean of the individual observation (gas production), μ = overall mean, Bi = block effect (replicate), Tj = treatment effect, and eij = residual error. Mean separations were performed using the Tukey test, and the significance was declared at *p* < 0.05. The principal component analysis was carried out using the PAST 4 software, version 4.11.

## 4. Conclusions and Recommendations

The crude extract yields of the four plant leaves used in this study increased with an increase in the amount of distilled water in the extraction solvents, as a replacement for methanol. *M*. *oleifera* crude extracts yields (g/10 g) increased from 3.24 to 3.92, *A. vera*, (2.35 to 3.11) *J. curcas* (1.77 to 2.26), and *P. betle* (2.42 to 3.53). However, the identified metabolites showed differing degrees of solubility unique to their plant leaves while most of the metabolite yields were unaffected by the extraction solvents. Although the *A. vera*, *J. curcas*, *M. oleifera*, and *P. betle* leaf extracts reduced the in vitro methane gas emission at the dosage of 50 mg kg^−1^ DM of *E. curvula* hay, decreasing the methane emission by 6.37–7.55%, 8.02–11.56%, 12.26–12.97, and 5.66–7.78, respectively, compared to the control treatment, the extraction solvents did not affect their methane reducing potential, total gas production, and organic matter digestibility. Furthermore, aloin A, aloin B and kaempferol (in *A. vera*), apigenin, catechin, epicatechin, kaempferol, tryptophan, procyanidins, vitexin-7-olate and isovitexin-7olate (in *J. curcas*), alkaloid, kaempferol, quercetin, rutin and neochlorogenic acid (in *M. oleifera)* and apigenin-7,4′-diglucoside, 3-*p*-coumaroylquinic acid, rutin, 2-methoxy-4-vinylphenol, dihydrocaffeic acid, and dihydrocoumaric acid (in *P. betle*) exhibited the methane reducing potential and hence, additional studies may be conducted to test the methane reducing properties of the individual metabolites, as well as their combined forms. Plant extracts could be more promising, and hence, further study is necessary to explore other extraction methods, as well as the encapsulation of extracts for the improved delivery of core materials to the target sites and to enhance methane reducing properties. Furthermore, the use of 70% aqueous extraction for the *Moringa oleifera* leaf is recommended, due to the reduced cost of the extractive solvents, lower cost and availability of *Moringa* plants in South Africa, especially in Gauteng Province. Finally, 70% aqueous-methanolic extractions of *Aloe vera*, *Jatropha curcas*, and *Piper betle* are also recommended for practical use in regions where they exist in abundance and are cost effective.

## Figures and Tables

**Table 1:** Effects of the solvent extraction on the yield of crude extracts (g/10 g) dry sample.

	70% CH_3_OH	85% CH_3_OH	100% CH_3_OH	SEM	*p* Value
*A. vera*	3.11 ^A^	2.42 ^B^	2.35 ^B^	0.10	0.009
*J. curcas*	2.26 ^A^	2.06 ^AB^	1.77 ^B^	0.05	0.006
*M. oleifera*	3.92 ^A^	3.38 ^B^	3.24 ^B^	0.12	0.013
*P. betle*	3.53 ^A^	3.07 ^A^	2.42 ^B^	0.14	0.002

SEM = standard error of means, *p* value = probability value, uppercase letters compare the means of the extract yield among all solvent extraction techniques of each plant material across the row. Means with different letters across the row for each parameter are significantly (*p* < 0.05) different.

**Table 2:** Identification and mean abundance of the phytochemicals (mg L^−1^) in *Aloe vera* extracts using 70%, 85%, and 100% aqueous-methanol.

RT (min)	Molecular Formula	Measured Mass (*m/z*)	Error *m/z* (ppm)	MS Fragment	UVmax (nm)	Compound	Classification of the Compounds	70%	85%	100%
10.94	C_16_H_18_O_8_	337.0928	−0.3	273,245,202	309	3-*p*-coumroyl quinic acid	Phenolic acid	7.65	8.42	2.40
11.64	C_19_H_22_O_9_	393.1185	−0.3	273245202	296	Aloesin	Chromone (*C*-glycosylated chromone)	59.51	281.46	293.30
14.02	C_27_H_30_O_15_	593.1526	−0.7	473,383,353	332	Aloe emodin-diglucosid	Anthrone (Anthracene compound)	5.04	30.51	25.99
15.15	C_27_H_30_O_15_	593.1455	−6.1	431,311,297,283,282,269	269,335	Aloe emodin-diglucoside isome	Anthrone (Anthracene compound)	28.45	92.67	91.18
16.25	C_21_H_20_O_11_	448.1304				Kaempferol-7-*O*-glucoside	Flavonoid (*C*-glycosylated flavoinoid)	8.32	6.50	5.22
16.66	C_21_H_22_O_10_	433.1138	1.6	313,270	304	10-hydroxyaloin B	Anthrone	1.45	60.38	74.93
16.89	C_21_H_22_O_10_	433.1133	0.9	313,270	305	10-hydroxyaloin A	Anthrone	0.91	58.23	70.58
18.61	C_24_H_26_O_12_	505.1343	−1.6	343,297,257	264,301	6-Malonylnataloin B (nataloin B)	Anthrone	3.04	77.87	83.30
19.24	C_24_H_26_O_12_	505.1355	−4.4	343,325,297,257	264,301	6-Malonylnataloin A (nataloin A)	Anthrone	1.97	55.97	59.22
20.16	C_29_H_30_O_12_	569.1669	0.2	407,243,161	300	Caffeoyl ester of aloesin	Chromone	3.14	43.32	44.84
21.01	C_21_H_22_O_9_	417.1194	0.0	297	297,354	Aloin B	Anthrone (Anthracene compound)	123.34	275.75	265.68
21.84	C_21_H_22_O_9_	417.1176	−2.4	297	297,354	Aloin A	Anthrone (Anthracene compound)	163.05	310.38	303.57

**Table 3:** Identification and the mean abundance of phytochemicals (mg L^−1^) in *Jatropha curcas* extracts using 70%, 85% and 100% aqueous-methanol.

RT (min)	Molecular Formula	Measured Mass (*m/z*)	Error *m/z* (ppm)	MS Fragment	UVmax (nm)	Compound	Classification of the Compounds	70%	85%	100%
7.73	C_45_H_38_O_18_	865.2037	3.7	577,407,289,125	279	Procyanidin trimer C1	Flavonoid	10.54	30.67	34.64
8.98	C_11_H_12_N_2_O_2_	203.0821	−0.3	149	279	Tryptophan	Amino acid	43.36	46.64	49.83
10.4	C_30_H_26_O_12_	577.1321	−4.9	407,289,125	279	Procyanidin dimer B	Flavonoid	96.38	104.34	109.56
10.78	C_30_H_26_O_12_	577.1322	−4.9	407,289,125	279	Procyanidin dimer B2	Flavonoid	49.05	95.19	99.05
11.06	C_15_H_14_O_6_	289.0712	−1.0	245,203,151,103	279	Catechin	Flavonoid	292.48	298.98	312.01
11.44	C_45_H_38_O_18_	865.1969	−1.3	577,407,289,125	279	Procyanidin trimer C2	Flavonoid	56.84	72.59	74.58
13.16	C_15_H_14_O_6_	289.0716	−1.4	245,203,151,103	279	Epicatechin	Flavonoid	101.19	125.93	143.53
15.47	C_26_H_28_O_14_	563.1395	−2.1	443,383,353	271,335	Apigenin-6-*C*-arabinosyl-8-*C*-arabinoside	Flavonoid (*C*-glycosylated flavoinoid)	141.07	137.38	137.52
15.69	C_21_H_20_O_11_	448.093	0.7	357,327,300	269,349	Kaempferol-7-*O*-glucoside	Flavonoid	7.89	6.31	6.37
16.91	C_21_H_20_O_10_	431.0966	−1.9	341,311,283	268,335	Vitexin-7-olate	Flavonoid (*C*-glycosylated flavoinoid)	142.42	143.18	144.79
17.44	C_21_H_20_O_10_	431.097	−2.1	341,311,283	271,335	Isovitexin-7-olate	Flavonoid (*C*-glycosylated flavoinoid)	188.89	187.05	190.77
19.81	C_27_H_30_O_14_	577.1558	0.7	269	267,335	Apigenin-7-*O*-rutinoside	Flavonoid (*O*-glycosylated flavoinoid)	78.32	77.60	80.49

RT = retention time.

**Table 4:** Identification and mean abundance of phytochemicals (mg L^−1^) in *Moringa oleifera* extracts using 70%, 85%, and 100% aqueous-methanol.

RT (min)	Molecular Formula	Measured Mass (*m/z*)	Error *m/z* (ppm)	MS Fragment	UVmax (nm)	Compound	Classification of the Compounds	70%	85%	100%
9.29	C_16_H_18_O_9_	353.0864	−2.5	191,179,135	325	neochlorogenic acid	Phenolic acid	203.93	205.09	198.07
10.85	C_16_H_18_O_8_	337.0922	−0.3	191,173,163	305	3-*p*-coumaroylquinic acid	Phenolic acid	108.21	110.85	107.67
11.72	C_16_H_18_O_9_	353.088	−1.4	191	325	chlorogenic acid	Phenolic acid	30.44	47.73	19.94
12.11	C_17_H_20_O_9_	367.1014	−1.9	193,134	323	Feruloylquinic aci	Phenolic acid	39.14	0	0
12.57	C_39_H_19_NO_7_	612.1063	−1	97	344	Alkaloid	Alkaloid	142.73	265.40	272.63
13.99	C_27_H_30_O_15_	593.1519	2.5	473,383,353,297	270,334	Kaempferol-3-*O*-rutinoside (isomer)	Flavonoid (*O*-glycosylated flavoinoid)	77.03	78.23	64.81
17.08	C_27_H_30_O_16_	609.1464	−0.3	300,271,255	256,354	Rutin	Flavonoid	102.18	105.07	101.61
17.56	C_21_H_20_O_12_	463.0873	−0.2	300,271,255	351	Quercetin-3-*O*-hexoside	Flavonoid (*O*-glycosylated flavoinoid)	94.24	94.18	89.58
18.36	C_23_H_22_O_13_	505.0983	0.2	300,271,255	354	Quercetin-3-*O*-(6″- acetyl-glucoside	Flavonoid (*O*-glycosylated flavoinoid)	84.60	82.54	86.07
18.71	C_27_H_30_O_15_	593.151	−1.9	285,271,255	265,348	Kaempferol-3-*O*-rutinoside	Flavonoid (*O*-glycosylated flavoinoid)	79.98	82.57	85.09
19.18	C_21_H_20_O_11_	448.0924	−0.7	285,255,227	265,348	Kaempferol-3-*O*-glucoside	Flavonoid (*O*-glycosylated flavoinoid)	146.74	146.99	157.28
20.31	C_23_H_22_O_12_	489.1047	3.5	285,255	265,348	Kaempferol-3-*O*-acetyl-glucoside	Flavonoid (*O*-glycosylated flavoinoid)	108.11	115.14	108.24

RT = retention time.

**Table 5:** Identification and mean abundance of phytochemicals (mg L^−1^) in the *Piper betle* extracts using 70%, 85%, and 100% aqueous methanol.

RT (min)	Molecular Formula	Measured Mass (m/z)	Error m/z (ppm)	MS Fragment	UVmax (nm)	Compound	Classification of Compounds	70%	85%	100%
7.45	C_15_H_18_O_10_	357.0825	0.8	345,195	326	Dihydrocaffeic acid 3′-*O*-β*D*-glucuronide	Phenolic acid	34.36	54.73	44.62
8.35	C_15_H_18_O_10_	357.0829	2.0	195,129,75	325	Dihydrocaffeic acid 4′-*O*-β*D*-glucuronide	Phenolic acid	25.14	27.52	21.13
9.91	C_15_H_18_O_9_	341.0859	0.6	195,163,119	312	Dihydro-*m*-coumaric acid 3′-*O*-β-*D*-glucuronide	Phenolic acid	98.09	101.57	88.52
10.39	C_15_H_18_O_9_	341.0862	−3.2	195.163	308	Dihydro-*p*-coumaric acid 4′-*O*-β-*D*-glucuronide	Phenolic acid	52.65	50.67	30.85
13.72	C_16_H_18_O_8_	337.0925	−0.3	191,173,163	305	3-*p*-coumaroylquinic acid	Phenolic acid	96.74	106.49	91.72
14.05	C_9_H_8_O_3_	163.04	−0.5	163	339	Coumaric acid	Phenolic acid	155.02	190.66	12.17
15.03	C_16_H_18_O_8_	337.0929	1.8	191,173,163	305	3-*p*-coumaroylquinic	Phenolic acid	128.56	129.21	105.34
15.51	C_27_H_30_O_16_	609.1448	1.8	489,429,357, 327,309	348	Rutin	Flavonoid	73.76	98.27	103.70
16.42	C_27_H_30_O_15_	593.1487	−1.7	413,293	268,334	Apigenin-7,4′-diglucoside	Flavonoid (*O*-glycosylated flavoinoid)	83.36	118.99	128.32
22.12	C_9_H_10_O_2_	149.0603				2-Methoxy-4-vinylphenol	Phenolic acid	675.93	775.91	814.11

RT = retention time.

**Table 6:** Methane, TGP, IVOMD and their relative estimation on Eragrostis hay, as affected by the plant extract yields of three different % solvent CH_3_OH:H_2_O (70, 85, and 100%) combinations.

Extract (50 mg kg^−1^ DM)	CH4 (mL g^−1^ DM)	TGP mL g^−1^ DM	CH4/TGP (×10^−3^)	IVOMD (g kg^−1^ DM)	TGP/IVOMD (mL kg^−1^ DM)	CH4/IVOMD (mL kg^−1^ DM)
Control	4.24A	166.50B	25.64A	608.41	273.97	6.96A
*Aloe vera* 70%	3.97B	170.49A	23.39B	607.40	280.91	6.54B
*Aloe vera* 85%	3.92B	167.73AB	23.49B	608.43	275.44	6.44B
*Aloe vera* 100%	3.94B	167.72AB	23.68B	604.08	277.10	6.53B
SEM	0.05	1.10	0.41	10.39	3.82	0.12
*p* value	0.01	0.04	0.01	0.97	0.35	0.04
Control	4.24A	166.50	25.64A	608.41	273.97	6.96A
*Jatropha curcas* 70%	3.79B	169.93	22.52B	616.17	275.20	6.15B
*Jatropha curcas* 85%	3.90B	172.30	22.78B	604.03	284.89	6.45B
*Jatropha curcas* 100%	3.75B	164.91	22.95B	608.37	271.01	6.16B
SEM	0.14	2.78	0.45	9.79	8.32	0.13
*p* value	0.04	0.13	0.003	0.63	0.41	0.04
Control	4.24A	166.50	25.64A	608.41	273.97	6.96A
*Moringa oleifera* 70%	3.70B	165.46	22.69B	608.67	271.95	6.09B
*Moringa oleifera* 85%	3.71B	165.23	22.73B	613.33	269.55	6.07B
*Moringa oleifera* 100%	3.69B	165.65	22.55B	613.00	270.02	6.02B
SEM	0.08	2.42	0.38	6.40	2.41	0.12
*p* value	0.002	0.95	0.001	0.87	0.35	0.001
Control	4.24A	166.50	25.64A	608.41	273.97	6.96A
*Piper betle* 70%	3.97B	168.42	23.65B	614.72	274.19	6.47B
*Piper betle* 85%	4.00B	171.89	23.41B	609.29	282.45	6.57B
*Piper betle* 100%	3.91B	166.36	23.66B	613.97	270.92	6.37B
SEM	0.06	2.18	0.43	6.08	3.87	0.09
*p* value	0.03	0.15	0.01	0.74	0.09	0.02

SEM: standard error of mean, CH_4_: methane TGP: total gas production, IVOMD: in vitro organic matter digestibility. A,B: The letters compare the means of extraction solvents for each plant species. The means with different letters within the column are significantly different (*p* < 0.05).

## Data Availability

Not applicable.

## Supplementary Materials

The following supporting information can be downloaded at: https://www.mdpi.com/article/10.3390/plants11233296/s1, Figures SA1: *Aloe vera* 70% CH3OH solvent extraction; Figure SA2: *Aloe vera* 85% CH3OH solvent extraction; Figure SA3: *Aloe vera* 100% CH3OH solvent extraction; Figure SP1: *Piper betle* 70% CH3OH solvent extraction; Figure SP2: *Piper betle* 85% CH3OH solvent extraction; Figure SP3: *Piper betle* 100% CH3OH solvent extraction; Figure SJ1: *Jatropha curcas* 70% CH3OH solvent extraction; Figure SJ2: *Jatropha curcas* 85% CH3OH solvent extraction; FigureSJ3: *Jatropha curcas* 100% CH3OH solvent extraction; Figure SM1: *Moringa oleifera* 70%CH3OH solvent extraction; Figure SM2: *Moringa oleifera* 85%CH3OH solvent extraction; Figure SM3: *Moringa oleifera* 100%CH3OH solvent extraction; Table S1: Correlation showing the effects of extraction solvents, extract yields and phytochemicals of plant extracts on CH4 emission, TGP and IVOMD of *Eragrostis curvula* hay. and Table S2: Principal component loadings of fermentation parameters of *Eragrostis curvula* hay fermented with three different aqueous-methanol (70, 85 and 100%) extractions of *Aloe vera, Jatropha curcas, Moringa oleifera* and *Piper betle* leaf extracts.
