# Supplementary material for: The Antimethanogenic Potentials of Plant Extracts: Their Yields and Phytochemical Compositions as Affected by Extractive Solvents
Source: Plants (Basel). 2022 Nov 29;11(23):3296. doi: 10.3390/plants11233296 (PMC9737672; doi:10.3390/plants11233296)
Supplement: Supplementary file 1 [file plants-11-03296-s001.zip › plants-2025822-supplementary.pdf]

plants

MDPI

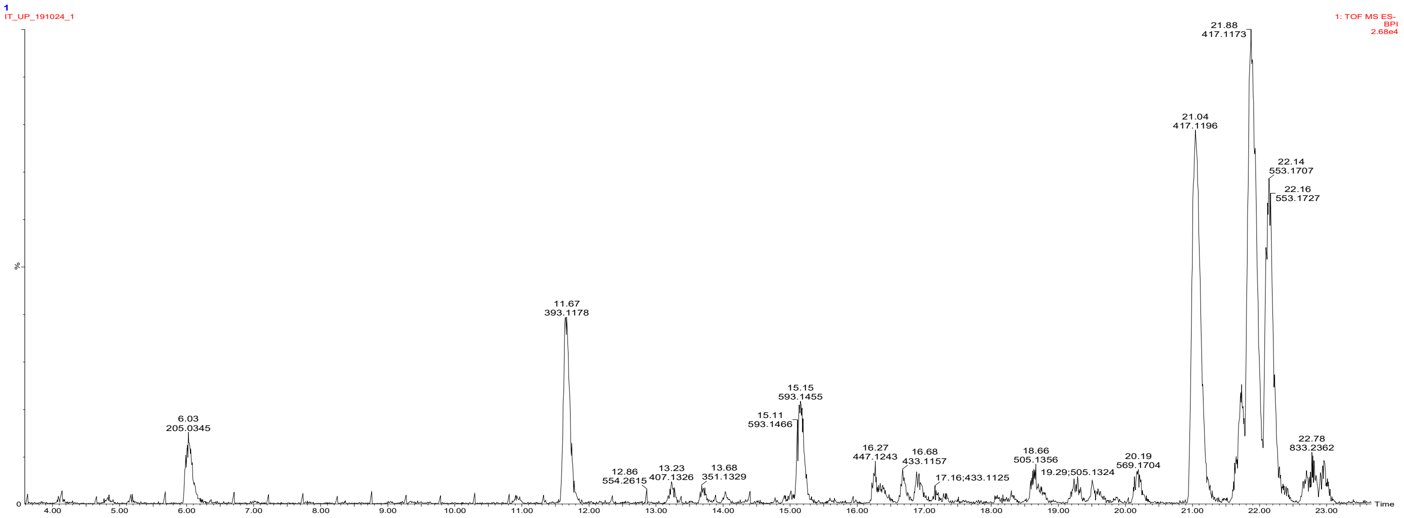

Figure SA1. *Aloe vera* 70% CH3OH solvent extraction.

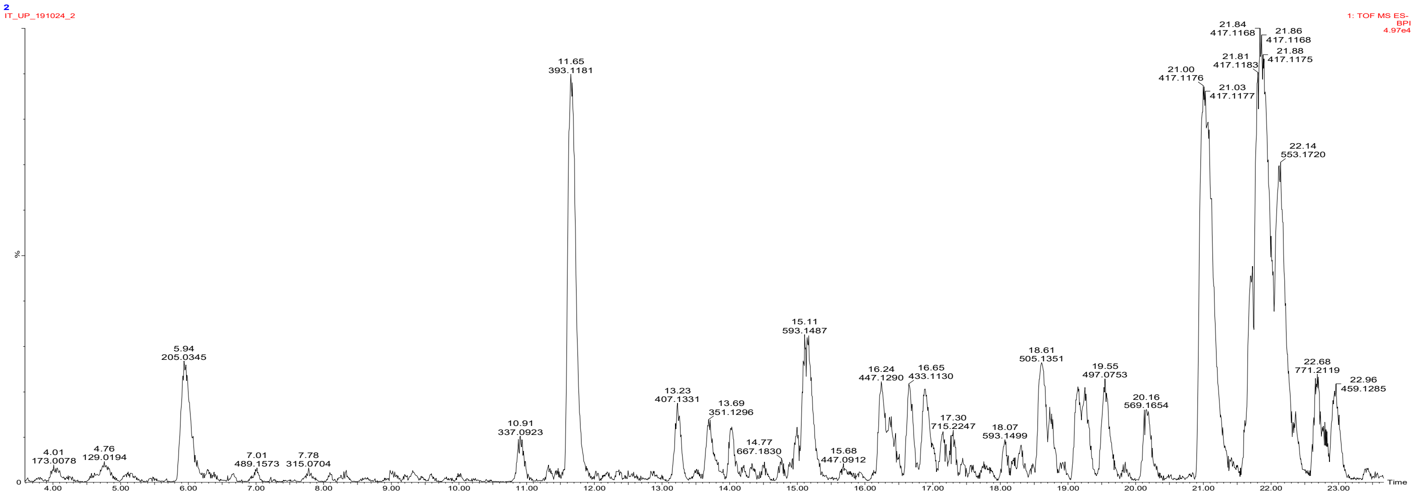

Figure SA2. *Aloe vera* 85% CH3OH solvent extraction.

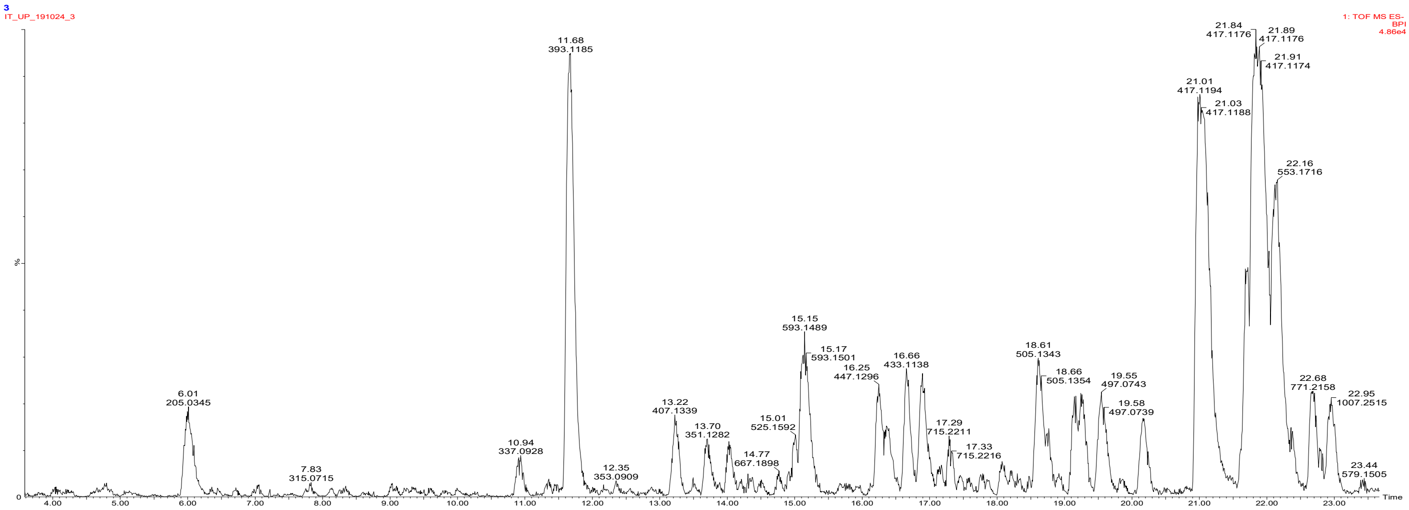

Figure SA3. *Aloe vera* 100% CH3OH solvent extraction.

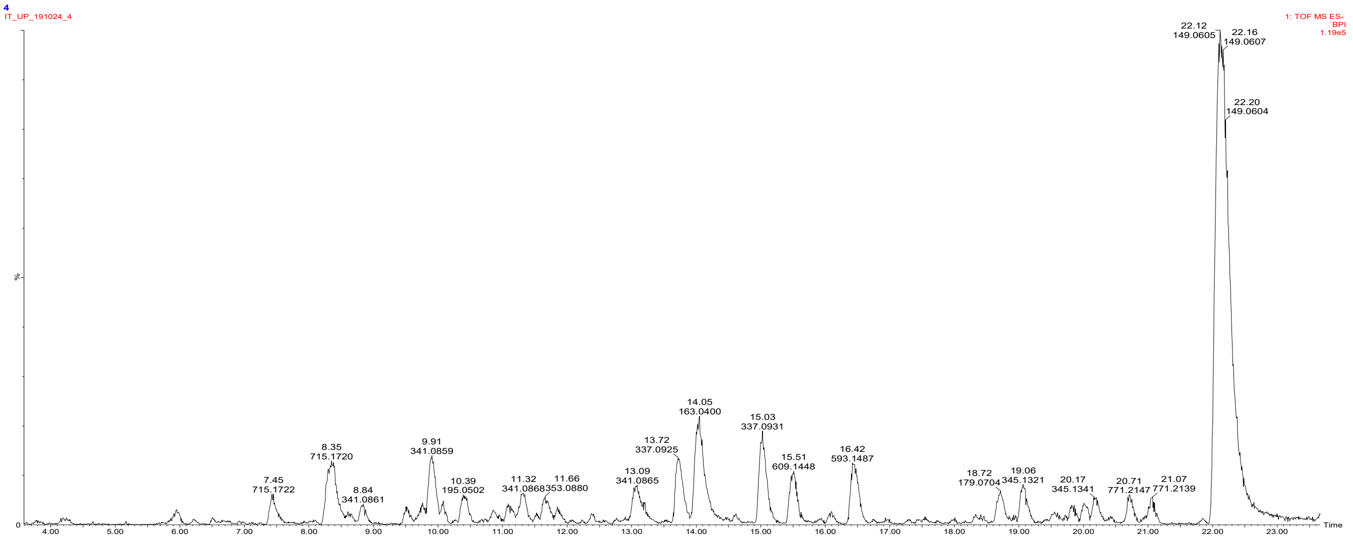

Figure SP1. *Piper betle* 70% CH3OH solvent extraction.

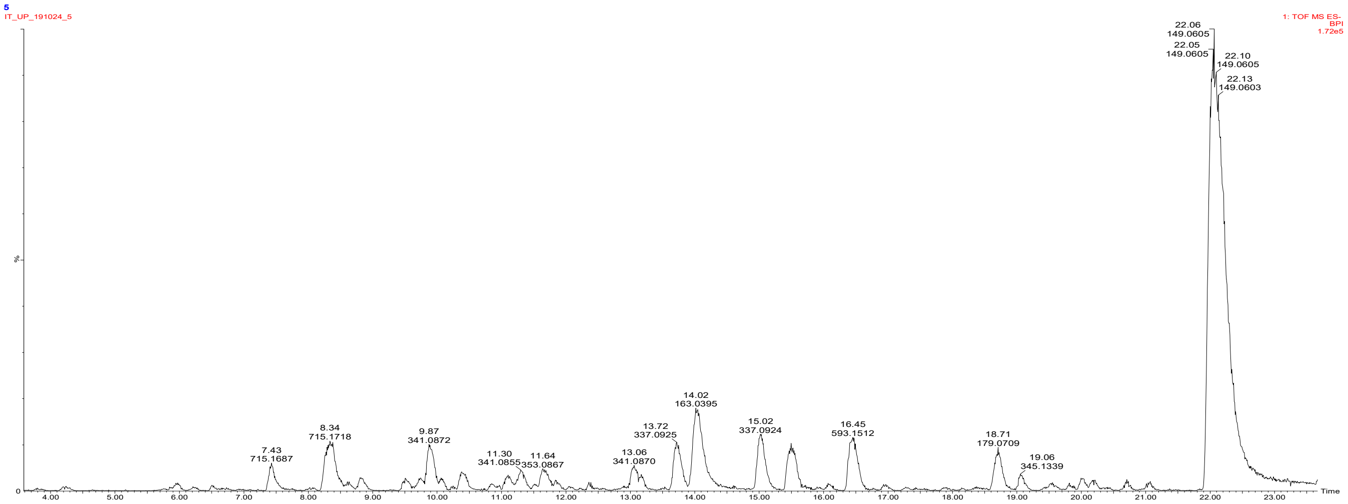

Figure SP2. *Piper betle* 85% CH3OH solvent extraction.

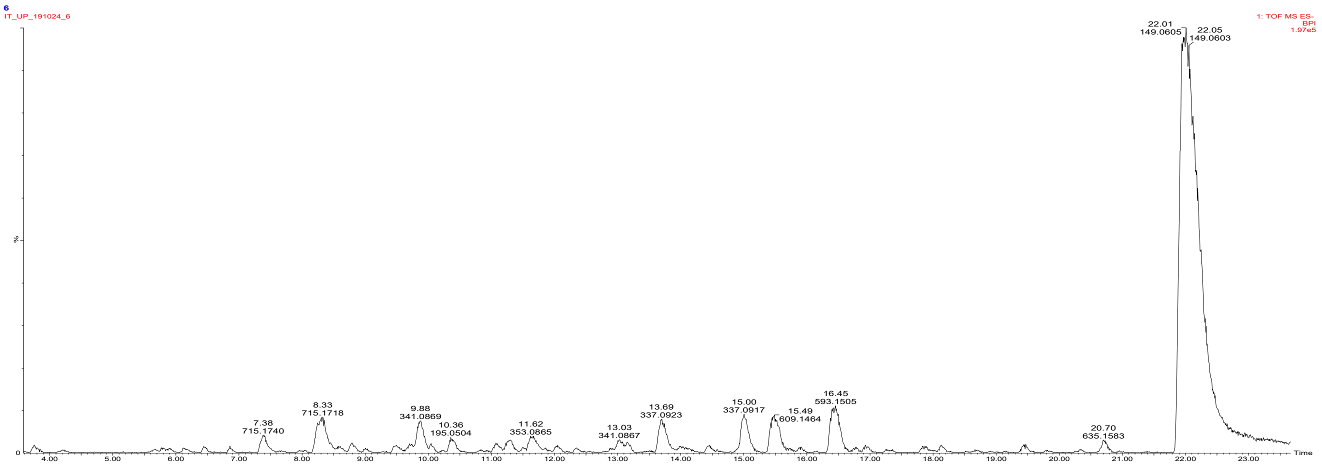

Figure SP3. *Piper betle* 100% CH3OH solvent extraction.

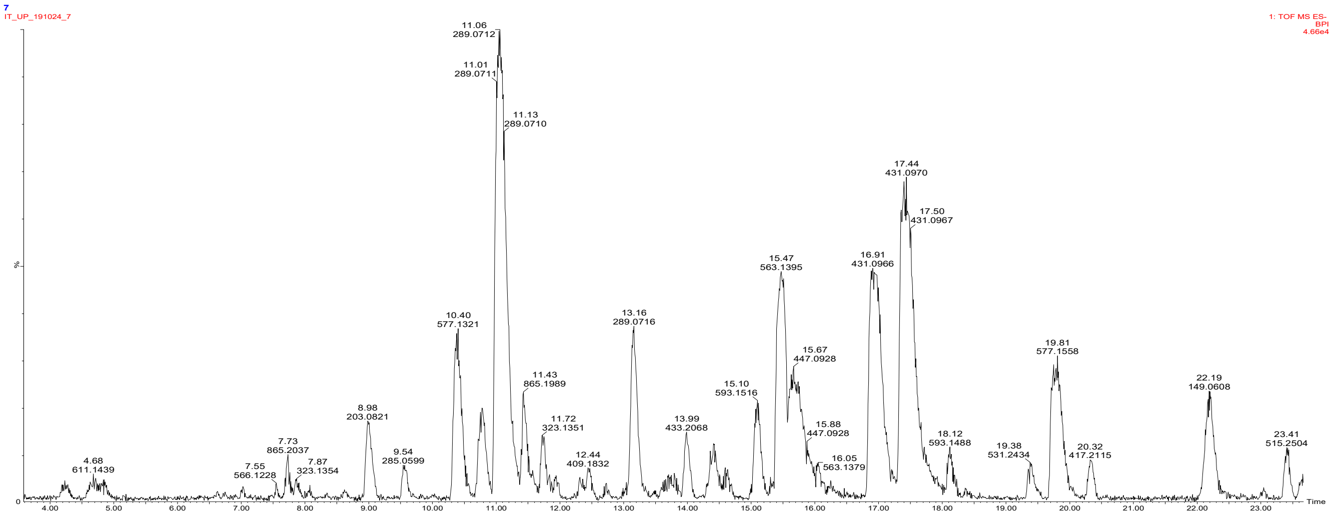

Figure SJ1. *Jatropha curcas* 70% CH3OH solvent extraction.

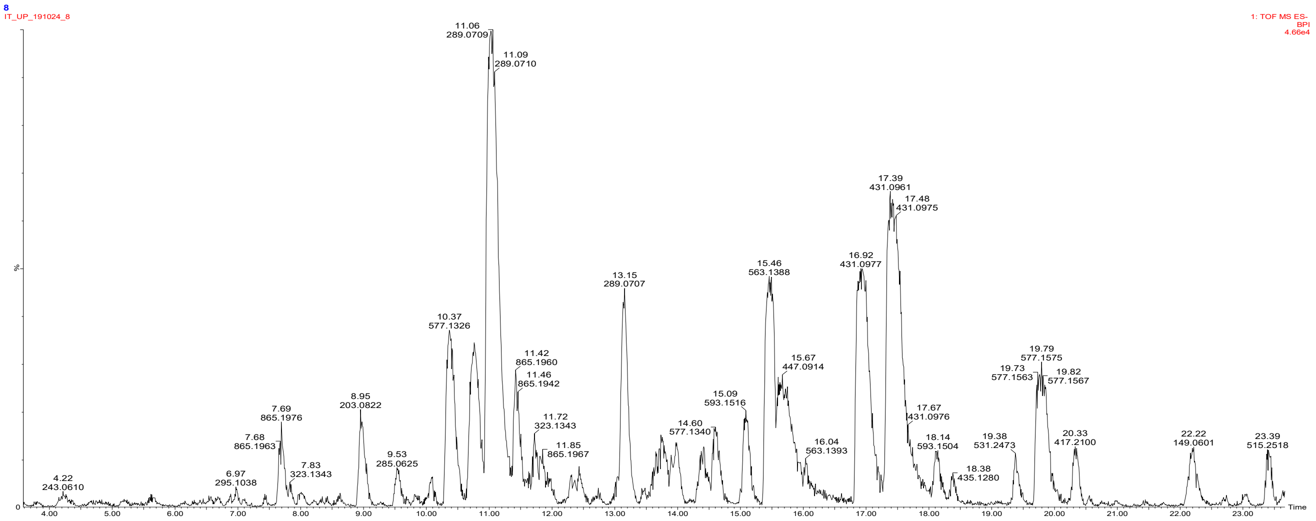

Figure SJ2. *Jatropha curcas* 85% CH3OH solvent extraction.

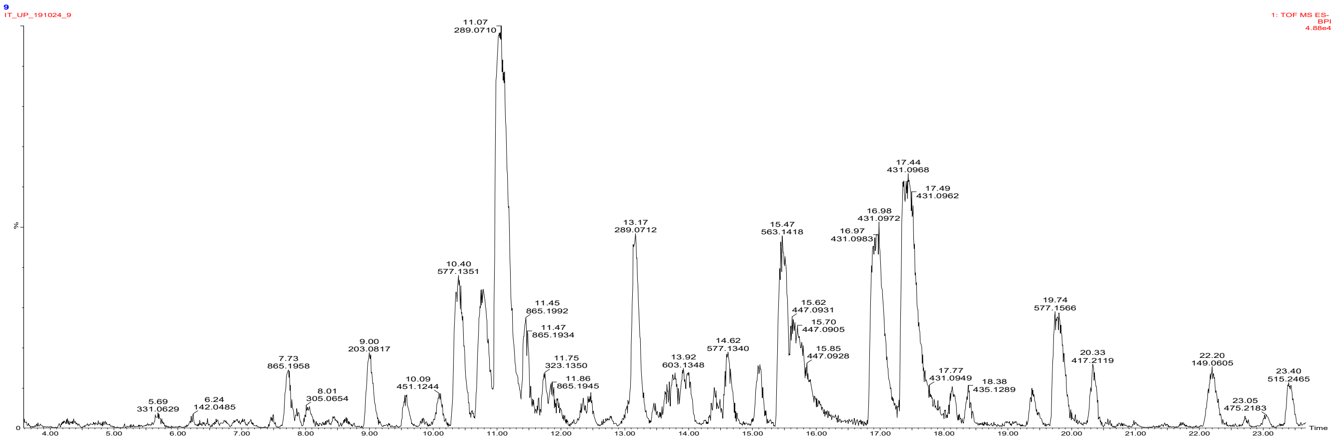

Figure SJ3. *Jatropha curcas* 100% CH3OH solvent extraction.

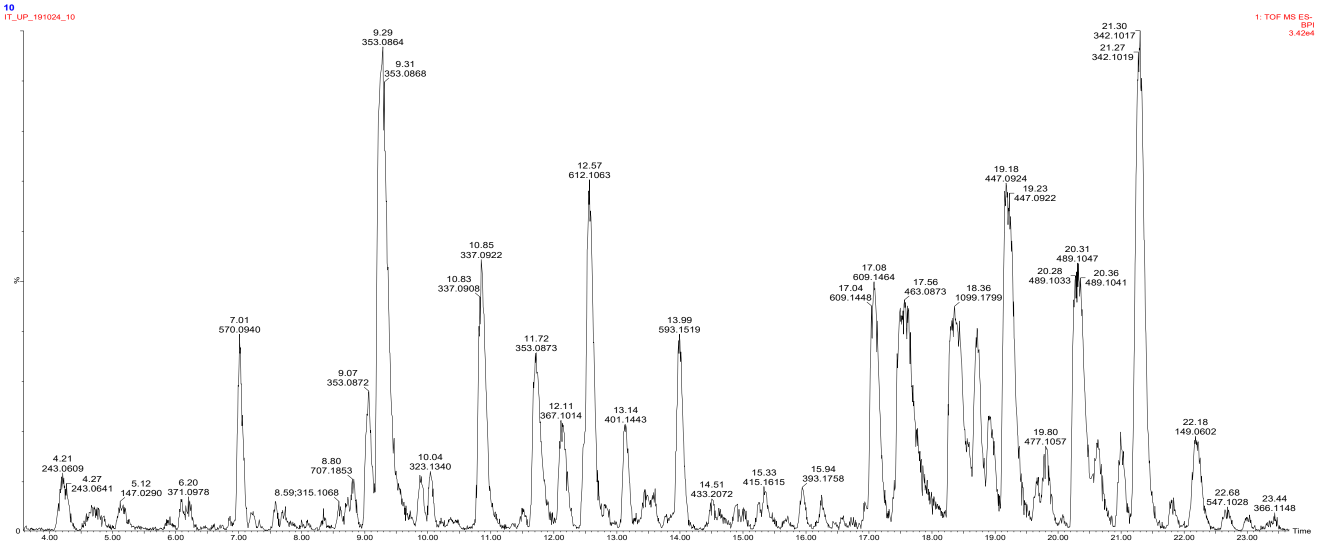

Figure SM1. *Moringa oleifera* 70%CH3OH solvent extraction.

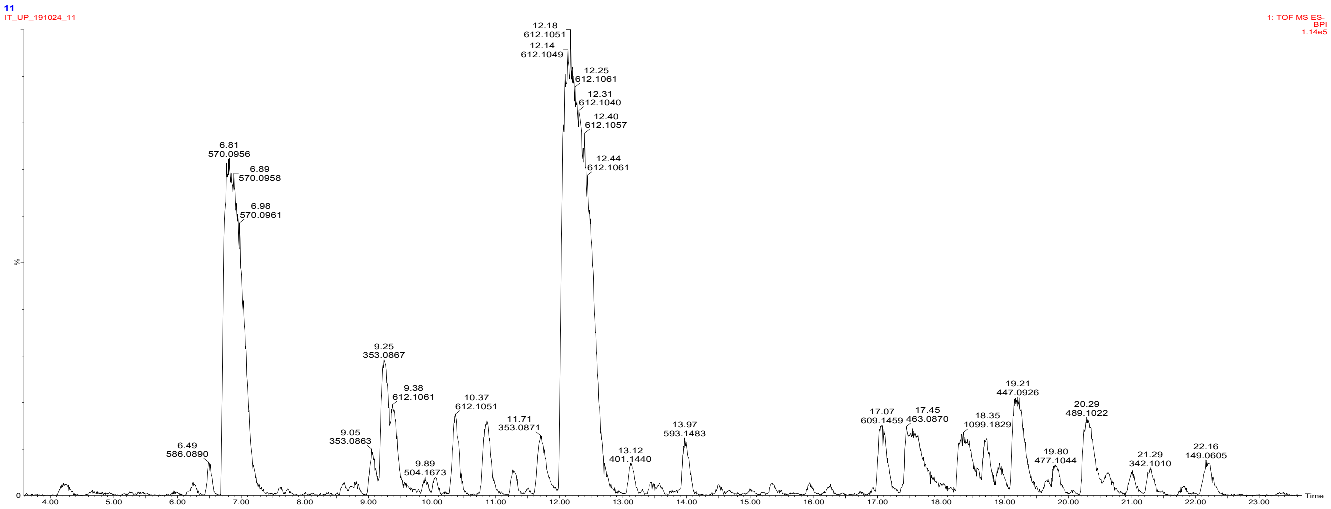

Figure SM2. *Moringa oleifera* 85%CH3OH solvent extraction.

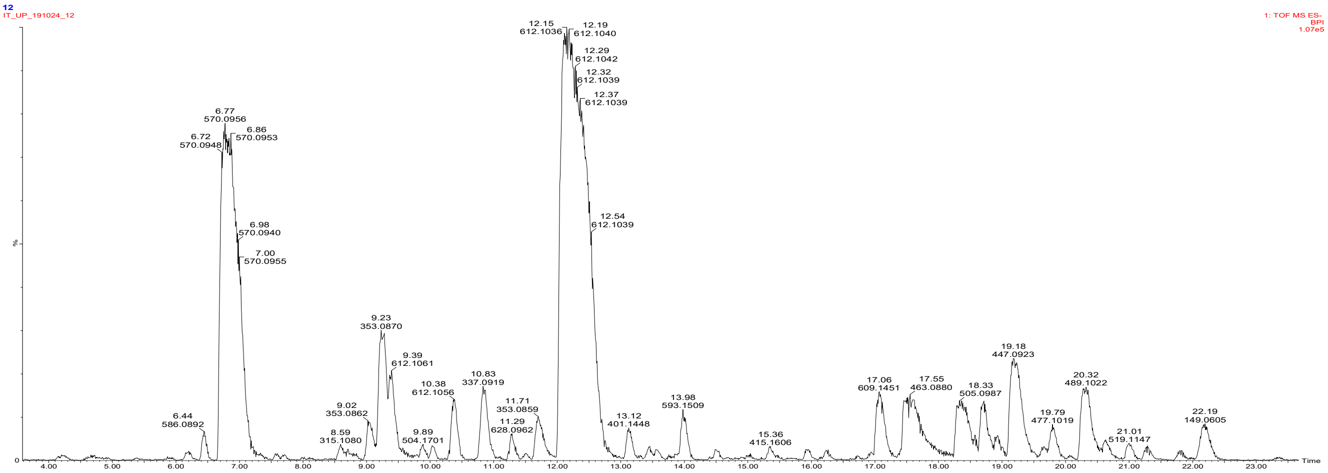

Figure SM3. *Moringa oleifera* 100%CH3OH solvent extraction.

Table S1. Correlation showing the effects of extraction solvents, extract yields and phytochemicals of plant extracts on CH4 emission, TGP and IVOMD of *Eragrostis curvula* hay.

Variable/metabolites	%CH4-Re	TGP	CH4/TGP	OMD	TGP/OMD	CH4/OMD	EY	
Aloe vera								
%CH4-Re (percentage methane reduction)	1							
TGP (total gas production)	.544	1						
CH4/TGP	-.980**	-.698	1					
OMD (organic matter digestibility)	-.385	-.071	.344	1				
TGP/OMD	.537	.973*	-.686	-.283	1			
CH4/OMD	-.997**	-.538	.977*	.316	-.514	1		
Extract Yield (EY)	-.750	.997*	-.815	.368	.927	.506	1	
Aloin A (AA)	.930*	.205	-.841	-.487	.226	-.924*	-.992*	-.992*
Aloin B (AB)	AB	.900*	.128	-.798	-.477	.149	-.895	-.990*
Aloe emodin (AE)	.815	-.041	-.685	-.461	-.018	-.811	-.990*	
Aloesin (Alos)	.771	-.104	-.633	-.516	-.063	-.762	-.999*	
Caffeoyl ester of aloesin (Ce)	.698	-.213	-.546	-.494	-.170	-.691	-.999*	
3-*p*-coumaroylquinic acid (Ci)	.769	.681	-.816	.262	.538	-.807	.470	
Hydroxyaloin A (HA)	.693	-.987	.861	-.444	-.893	-.433	-.997*	
Hydroxyaloin B (HB)	.654	-.246	-.499	-.591	-.174	-.637	-.995*	
Kaempferol hexoside	.901*	.845	-.967*	-.193	.805	-.904*	.943	
Nataloin A (NA)	.677	-.239	-.521	-.502	-.193	-.668	-.999*	
Nataloin B (NB)	.678	-.237	-.522	-.509	-.189	-.669	1.00**	
Jatropha curcas								
%CH4-Re (percentage methane reduction)	1							
TGP (total gas production)	.131	1						
CH4/TGP	-.947*	-.443	1					
OMD (organic matter digestibility)	.271	-.101	-.197	1				
TGP/OMD	-.031	.910*	-.274	-.503	1			
CH4/OMD	-.992**	-.109	.930*	-.391	.098	1		
Extract Yield (EY)	-.358	.740	-.975	.549	.393	.076	1	
Apigenin (Api)	.960*	.386	-.995**	.133	.249	-.935*	.788	
Catechin (Ca)	.964*	.342	-.985**	.085	.230	-.932*	-.996*	
Epicatechin (Epi)	.935*	.220	-.923*	-.084	.193	-.883	-.980	
Kaempferol hexoside	.955*	.406	-.994**	.304	.198	-.952*	.788	
Procyanidin dimer B1 (PDB1)	.957*	.325	-.975*	.035	.235	-.920*	-.975	
Procyanidin dimer B2 (PDB2)	.809	.211	-.810	-.345	.295	-.729	-.847	
Procyanidin trimer C1 (PTC1)	.716	.086	-.687	-.470	.239	-.623	-.889	
Procyanidin trimer C2 (PTC2)	.923*	.309	-.940*	-.095	.275	-.870	-.864	
Tryptophan (Try)	.960*	.311	-.973*	.038	.222	-.923*	-.994*	
Vitexin-7-olate (V)	.960*	.372	-.991**	.104	.248	-.931*	-.995*	
Isovitexi-7-olate (Ivi)	.963*	.367	-.992**	.116	.240	-.935*	-.594	
Moringa oleifera								
%CH4-Re (percentage methane reduction)	1							
TGP (total gas production)	-.936*	1						
CH4/TGP	-1.000**	.935*	1					
OMD (organic matter digestibility)	.597	-.647	-.601	1				
TGP/OMD	-.857	.853	.860	-.925*	1			

CH4/OMD	-.999**	.936*	1.000**	-.626	.875	1	
Extract Yield (EY)	-.454	-.224	.558	-.858	.893	.885	1
Alkaloids (A)	.979*	-.943*	-.977*	.458	-.756	-.971*	.997*
chlorogenic acid (Cl)	.793	-.956*	-.791	.669	-.797	-.795	.140
3-*p*-coumaroylquinic acid (Ci)	.998**	-.958*	-.997**	.612	-.864	-.997**	-.083
Feruloylquinic acid (F)	.325	-.301	-.318	-.530	.191	-.288	.961
Kaempferol (K)	.998**	-.955*	-.998**	.616	-.867	-.998**	-.534
neochlorogenic acid (N)	.997**	-.959*	-.997**	.597	-.854	-.996**	.605
Quercetin (Q)	.998**	-.952*	-.998**	.590	-.851	-.997**	.999*
Rutin (R)	.997**	-.959*	-.997**	.613	-.865	-.997**	-.088
Piper betle							
%CH4-Re (percentage methane reduction)	1						
TGP (total gas production)	.299	1					
CH4/TGP	-.938*	-.608	1				
OMD (organic matter digestibility)	.769	-.236	-.576	1			
TGP/OMD	-.046	.937*	-.295	-.549	1		
CH4/OMD	-.997**	-.229	.912*	-.817	.120	1	
Extract Yield (EY)	-.726	.395	-.133	.029	.368	.583	1
Apigenin	.944*	.495	-.948*	.516	.195	-.916*	-.911
chlorogenic acid (Cl)	.629	.831	-.842	.314	.617	-.592	.854
3-*p*-coumaroylquinic acid (Ci)	.918*	.626	-.995**	.588	.315	-.893	.772
Coumaric acid (Cu)	.389	.894	-.668	.078	.767	-.344	.817
Dihydrocaffeic acid (Dca)	.893	.692	-.986**	.440	.408	-.855	-.170
Dihydrocoumaric acid (Dcu)	.892	.646	-.985**	.581	.340	-.869	.894
Methoxy-4-vinylphenol (Met)	.972*	.506	-.986**	.612	.184	-.951*	-.939
Rutin (R)	.954*	.510	-.966*	.543	.203	-.927*	-.900

*correlated at p<0.05, **correlated at p<0.01

Table S2. Principal component loadings of fermentation parameters of *Eragrostis curvula* hay fermented with three different aqueous-methanol (70, 85 and 100%) extractions of *Aloe vera, Jatropha curcas, Moringa oleifera* and *Piper betle* leaf extracts.

Variables/metabolites	PC 1×100	PC 2×100
Aloe vera		
%CH4-Re (percentage methane reduction)	1.14	-3.79
TGP (total gas production)	0.02	-3.44
CH4/TGP	-0.30	1.50
OMD (organic matter digestibility)	-0.39	-0.51
TGP/OMD	0.07	-5.65
CH4/OMD	-0.08	0.26
EY (Extract yield)	-0.08	-0.57
Aloin A (Aa)	54.87	-52.16
Aloin B (Ab)	49.59	-26.85
Aloe emodin (AE)	23.27	8.44
Aloesin (Alos)	56.85	44.60
Caffeoyl ester of aloesin (Ce)	9.07	12.60
3-*p*-coumaroylquinic acid (Ci)	0.68	-6.55
Hydroxyaloin A (HA)	6.94	50.54
Hydroxyaloin B (HB)	14.13	24.68

Kaempferol hexoside	0.74	-6.24
Nataloin A (NA)	11.98	18.75
Nataloin B (NB)	16.75	26.10
% Variance	96.71	3.24
Jatropha curcas		
%CH4-Re (percentage methane reduction)	2.21	-2.00
TGP (total gas production)	0.50	-3.81
CH4/TGP	-0.62	0.96
OMD (organic matter digestibility)	0.13	-15.76
TGP/OMD	0.63	1.67
CH4/OMD	-0.15	0.29
EY (Extract yield)	-0.006	-0.62
Apigenin (ApiA)	30.30	-23.40
Catechin (Cat)	66.18	-14.27
Epicatechin (Epi)	27.62	42.68
Procyanidin dimer B1 (PDB1)	22.82	5.27
Procyanidin dimer B2 (PDB2)	18.45	69.47
Procyanidin trimer C1 (PTC1)	5.87	33.86
Procyanidin trimer C2 (PTC2)	15.13	19.30
Tryptophan (Try)	10.29	2.70
Vitexin (Vit)	31.43	-15.22
Iso-vitexin (Ivi)	41.37	-23.11
% Variance	98.33	1.53
Moringa oleifera		
%CH4-Re (percentage methane reduction)	2.07	-1.12
TGP (total gas production)	-0.17	0.08
CH4/TGP	-0.49	0.30
OMD (organic matter digestibility)	0.46	-6.13
TGP/OMD	-0.56	3.43
CH4/OMD	-0.15	0.13
EY (Extract yield)	0.004	0.81
Alkaloids (A)	51.85	68.61
3-*p*-coumaroylquinic acid (Ci)	17.93	-9.11
Chlorogenic acid (Cl)	5.45	-5.51
Feruloyl quinic acid (F)	2.41	57.12
Kaempferol (K)	68.56	-40.05
Neochlorogenic acid (N)	33.34	-10.47
Quercetin hexoside (Q)	29.16	-8.61
Rutin (R)	16.95	-8.83
% Variance	98.78	1.08
Piper betle		
%CH4-Re (percentage methane reduction)	0.32	-0.76
TGP (total gas production)	0.32	1.91
CH4/TGP	-0.25	-0.14
OMD (organic matter digestibility)	0.46	-1.03
TGP/OMD	0.26	4.19
CH4/OMD	-0.06	0.07
EY (Extract yield)	-0.01	0.46
Apigenin (Api)	13.74	-10.53
3-*p*-coumaroylquinic acid (Ci)	26.13	24.78
Chlorogenic acid (Cl)	1.16	4.48

Coumaric acid (Cu)	13.60	90.52
Dihydrocaffeic acid (Dca)	8.48	5.29
Dihydrocoumaric acid (Dcu)	16.65	21.52
Methoxy-4-vinylphenol (Met)	91.99	-22.53
Rutin (R)	11.37	-6.23
% Variance	95.56	4.27
